# A cross-sectional survey of parental perceptions of COVID-19 related hygiene measures within schools and adherence to social distancing in journeys to and from school

**DOI:** 10.1136/bmjpo-2020-000825

**Published:** 2020-09-09

**Authors:** Louise E Smith, Lisa Woodland, Richard Amlôt, Antonia Rubin, G James Rubin

**Affiliations:** 1 Department of Psychological Medicine, King's College London, London, UK; 2 Emergency Response Department, Public Health England, Salisbury, UK; 3 Weald of Kent Grammar School, Tonbridge, UK

**Keywords:** adolescent Health, psychology

## Abstract

During the early stages of the COVID-19 pandemic, schools in England were only kept open to children of ‘key workers’ and, from 1 June, to children in reception, year 1 and year 6. Our cross-sectional survey of parents found low rates of attendance (<50%) in both groups. Many parents whose children attended school reported low adherence to hygiene measures (eg, not maintaining distance from others during school drop-off) and doubted that their school was adhering to COVID-19 guidelines. This must be improved if parents are to feel confident about a more comprehensive return to school, as planned for September.

The COVID-19 pandemic has led to schools closing across the world, with substantial psychological and educational costs.^1^ Reopening schools when safe to do so is a priority. In England, schools were kept open to children of ‘key workers’. Children in reception (aged 4–5 years), year 1 (5–6 years) and year 6 (10–11 years) were encouraged to return to school from 1 June 2020. The return was voluntary for both groups, and the Government required schools to put measures in place to reduce the likelihood of disease transmission.^2^ These included: frequent hand cleaning; children mixing in groups of 15 or fewer; maintaining physical distancing where possible; minimising parent contact at the school gates; and limiting use of public transport. Additional steps were also recommended by some schools, including advising parents to wash children’s clothes daily and asking children to have their temperature taken daily. Protective measures are important both to reduce infection rates and to give parents confidence that it is safe for their children to return. If parents report that infection control measures are not in place, then this should be taken as a warning that hygiene practices need to be improved, that communication with parents needs to be improved or both.

BMG Research^3^ recruited 2447 participants on our behalf using active sampling from its existing panel to conduct an on-line poll to investigate the parental perception of the measures being implemented in schools and the number of physical contacts parents had while taking children to and from school. Four hundred thirty-seven participants were excluded (dropouts, non-eligibility and quality control). Participants were paid on average £0.60. Data were collected from 8 to 10 June, 1 week after schools opened to children in reception, year 1 and year 6. The sample fell within five percentage points of the known distribution of school children in England by child gender, school year and school they attended (fee paying or state-funded).^4^

The research was approved by the King’s College London Psychiatry, Nursing and Midwifery Research Ethics Subcommittee (LRS-19/20-18787).

## Patient and public involvement

A school trustee assisted in designing the questionnaire and coauthored this report.

A total of 2010 parents completed the survey in full (16 responses were coded as missing data because of sample grouping inconsistencies): 621 did not have a child eligible for school; 803 had children in reception, year 1 or year6 (‘eligible year groups’); 570 parents reported that they or their spouse was a key worker and that they did not have a child in an eligible year group. Of children in eligible year groups, only 370 (46%) had attended school for at least 1 day in the past week and 432 (54%) had not (n=1 ‘do not know’). Of children of key worker parents, only 72 (13%) had attended school and 497 (87%) had not (n=1 ‘do not know’). The experiences and perceptions of parents whose child had attended school are presented in table 1. Parents reported suboptimal levels of almost every hygiene practice that we asked about, including limited handwashing facilities on the way into schools or classrooms, class sizes that breached the recommended limit of 15 and not maintaining physical distance from other people during the school run.

**Table 1:** Experience and perceptions of parents (n=442/1371) in England whose child had attended school in the past week (data collection: 8 to 10 June 2020)

Item stem	Survey item wording (participants were asked to ‘tick any that apply’)	Number (%) of parents of children in reception, year 1 and year 6 responding ‘yes’ to the item (n=370)	Number (%) of key worker parents of children in other year groups responding ‘yes’ to the item (n=72)
Thinking about the facilities or procedures at your child’s school. Which of the following, if any, are actually happening as far as you are aware?	There are hand washing facilities or hand gel dispensers at the entrance to the school that are working	178 (48)	36 (50)
	There are hand washing facilities or hand gel dispensers at the entrance to the classrooms that are working	197 (53)	36 (50)
	Children’s hand washing or hand gel use is being monitored at school	211 (57)	37 (51)
	My child’s class size is now 15 or fewer	228 (62)	43 (60)
	The school has used markings or barriers to help children keep their distance from each other	191 (52)	38 (53)
	Children are having their temperature checked on the way in	106 (29)	19 (26)
In this question, we are interested in things that happened on the most recent day that your child went to school. Please remember that this survey is anonymous—please be honest in your answers.	Either on the way to or from school, or at the school gates, I (had physical contact with someone that I don’t live with OR was within 1 m of someone I do not live with for 1 min or longer OR I was between 1 and 2 m of someone that I do not live with for 15 min or longer)*	145 (39)	22 (31)
	My child used public transport to get to or from school	44 (12)	7 (10)
	My child shared a lift in a car with another family to get to or from school	42 (11)	5 (7)
	I gave a lift in a car to a child from another family to get them to or from school	41 (11)	9 (12.5)
	My child (washed their hands OR had a shower or bath) as soon as they got home from school*	258 (70)	50 (69)
	I washed my child’s clothes after they got home from school	169 (46)	26 (36)

*Data from multiple items have been combined for presentation.

†Key worker parents with children in reception, year 1 or year 6 are excluded from this group.

Given the urgent need for information, our data are based on a non-probability sample generated according to market research best practice and therefore care is required in interpretation because of likely selection bias.^5^ Nonetheless, the results are noteworthy. If school attendance is to increase, then parents must have confidence that good hygiene practices are in place. Urgent action is required to provide this by schools assuring parents of the hygiene measures that have been implemented and reiterating the social distancing guidance.

## Supplementary Material

Author's manuscript
